# Determinants of timely initiation of breastfeeding among mothers in Goba Woreda, South East Ethiopia: A cross sectional study

**DOI:** 10.1186/1471-2458-11-217

**Published:** 2011-04-08

**Authors:** Tesfaye Setegn, Mulusew Gerbaba, Tefera Belachew

**Affiliations:** 1 College of Public Health and Medical Sciences, Department of Population and Family Health, Jimma University, Jimma, Ethiopia; 2 College of Health Sciences, Department of Nursing, Madawalabu University, Bale Goba, Ethiopia

## Abstract

**Background:**

Although breastfeeding is universal in Ethiopia, ranges of regional differences in timely initiation of breastfeeding have been documented. Initiation of breastfeeding is highly bound to cultural factors that may either enhance or inhibit the optimal practices. The government of Ethiopia developed National Infant and Young Child Feeding Guideline in 2004 and behavior change communications on breast feeding have been going on since then. However, there is a little information on the practice of timely initiation of breast feeding and factors that predict these practices after the implementation of the national guideline. The objective of this study is to determine the prevalence and determinant factors of timely initiation of breastfeeding among mothers in Bale Goba District, South East Ethiopia.

**Methods:**

A community based cross sectional study was carried out from February to March 2010 using both quantitative and qualitative methods of data collection. A total of 608 mother infant pairs were selected using simple random sampling method and key informants for the in-depth interview were selected conveniently. Descriptive statistics, bivariate analysis and multivariable logistic regression analyses were employed to identify factors associated with timely initiation of breast feeding.

**Results:**

The prevalence of timely initiation of breastfeeding was 52.4%. Bivariate analysis showed that attendance of formal education, being urban resident, institutional delivery and postnatal counseling on breast feeding were significantly associated with timely initiation of breastfeeding (P < 0.05). After adjust sting for other factors on the multivariable logistic model, being in the urban area [AOR: 4.1 (95%C.I: 2.31-7.30)] and getting postnatal counseling [AOR: 2.7(1.86-3.94)] were independent predictors of timely initiation of breastfeeding.

**Conclusions:**

The practice of timely initiation of breast feeding is low as nearly half the mothers did not start breastfeeding with one hour after delivery. The results suggest that breast feeding behavior change communication especially during the post natal period is critical in promoting optimal practice in the initiation of breast feeding. Rural mothers need special attention as they are distant from various information sources.

## Background

Exclusive breastfeeding, preceded by timely initiation and appropriate complementary feeding practices are universally accepted as essential elements for the satisfactory growth and development of infants and for prevention of childhood illness [1-3]. Timely initiation of breastfeeding is defined as putting the newborn to the breast within one hour of birth. Timely initiation of breastfeeding is not only the easiest, cost effective and most successful intervention; it also tops the table of life-saving interventions for the health of the newborn [4-6]. Twenty two percent of neonatal deaths could be prevented, if all infants are put to the breast within the first hour of birth [7].

The prevalence of timely initiation of breastfeeding in some developing countries other than Ethiopia was documented as in Ghana (41%), Sudan (54.2%), Zambia; (70%), Jordan (49.5%), North Jordan (86.6%), Nepal (72.2%), Bolivia; (74%). In Ethiopia, one third of babies do not receive breastfeeding within the first hour of birth and national prevalence of breastfeeding initiation was documented to be low with wide regional differences [1,8-14].

Realizing the importance of timely initiation of breastfeeding, the Ethiopian government had developed infant and young child feeding guidelines giving appropriate emphasis to key messages on timely initiation of breastfeeding in 2004 [15]. Since then, different interventions like breast feeding promotions have been given at health institutions and at the community level by community health extension workers and other health care providers. However, these efforts are not based on systematic evidence on the level of existing practice which might be due to the paucity of data from studies on timely initiation. Therefore, the study sets out to address the issues related to prevalence of timely initiation of breastfeeding and associated factors among mothers in Goba Woreda, Ethiopia.

## Methods

### Study setting and Sample

A community-based cross-sectional study using both quantitative and qualitative methods of data collection was conducted in Goba district of Bale Zone in the south Eastern Ethiopia. Bale zone is the second largest Zones in Oromia regional state with an area of 67.329.6 km^2 ^and located 430 km from the capital, Addis Ababa. The temperature ranges from 3.5-32°c. The area is known by its eye catching natural and tourist appealing topography like Bale Mountains and Souf-Umor cave. Goba woreda is one of the 20 woreda in Bale zone having both rural and urban population. The district has one hospital, one health center and more than 20 health posts. According to Goba District Health Office report, the estimated total number of infants in the woreda is 1923 by the year 2009.

The sample size was determined using single population proportion formula assuming an expected prevalence for timely initiation of breast feeding of 50% with a finite population correction. The sample was multiplied by a design effect of 2 and by 10% was added non response it giving the final sample size of 668. A total of 668 mother-infant pairs were selected using stratified cluster sampling technique from the urban and rural residences then census was conducted to get the sampling frame for selecting mother- infant pairs by simple random sampling technique. For the in-depth interview, 23 individuals (6 health care providers, 9 breastfeeding mothers-three urban and six rural and eight community health extension workers) were selected conveniently.

### Measurements

Quantitative data were gathered using a questionnaire adapted from Ethiopian Demographic Health Survey, WHO, and LINKAGE project which were designed to assess infant and young child feeding practices in developing countries including Ethiopia [8,14,16]. The questionnaire was translated and contextualized to suit the local situation. Data on socio-demographic and economic factors (income, level of education etc), obstetric factors (like birth intervals, parity, Ante natal care) and health service related factors/practices including post natal counseling were collected by interviewing the mothers of index children. A semi-structured open-ended interview guide was used as a guide for the key informant interview.

The data were collected by 12th grade complete students who took an intensive training for two days was given on the questionnaire and on general approaches to data collection. Timely initiation of breastfeeding was measured by asking mothers provide information regarding the time at which their index infant was put to the breast after delivery. The prevalence of timely initiation of breastfeeding was calculated as the ratio of infants below one year put to the breast within 1 hour of delivery to the total number of infants (< 12 months).

### Data analysis

Quantitative data were checked for completeness, inconsistencies, entered, coded, cleaned and analyzed using SPSS for windows version 16.0 (SPSS Inc. version 16.1, Chicago, Illinois). Descriptive statistics was computed to determine the prevalence of timely initiation of breastfeeding. Proportions were compared by timely initiation of breast feeding using Chi-square tests. First a bivariate logistic regression was performed. Variables that showed significant association with timely initiation of breastfeeding in the bivariate models were entered in a multivariable logistic model. To identify independent predictors of timely initiation of breastfeeding, a multivariable logistic regression model with timely initiation of breast feeding as dependent variable was constructed. All tests were two-sided and a P < 0.05 was considered statistically significant. We present the results as proportions, output of the logistic regression as Odds Ratios (OR) with 95% Confidence Intervals. The qualitative data were transcribed in to English language and analyzed manually. Data captured using the field notes were transcribed verbatim into English language and double checked by the investigator. The transcribed data were read carefully and categorized in to thematic frameworks and color coded. The data were summarized by thematic areas and presented in narratives triangulated with the quantitative data. Well-said verbatim of the study participants were used as illustrations in presenting the results.

Letter of Ethical approval was received from Jimma university ethical clearance committee. Official letter of co-operation was also obtained from Oromia Health Bureau, Zonal Health Desk & Woreda Health Office. Informed verbal consent was secured from study participants in their own language explaining the purpose of the study, potential risk and benefits of partaking in the study and the right to with draw from the study any time. The participants were also assured about the confidentiality of the data.

## Results

Out of 668 mother-infant pairs sampled, 608 were included in the analysis making the response rate 91.01%. The mean (± SD) age of mothers was 26.5 (± 5.5) years. Thirty six percent of mothers were in the age group of 25-29 years. Sixty nine percent of respondents were Muslims by religion. The largest ethnic group was Oromo 542 (89.1%) followed by Amhara 60(9.9%). Concerning the educational status of mothers, 371(61.0%) had attended formal school of which 275(45.2%) completed primary school (grade1-8). Sixteen percent of the study subjects completed secondary school (9^th ^grade and above). Majority of the mothers were married 579(95.7%) and house wife by occupation 498(82.3%). Four hundred eighty-eight (80.3%) of the study participants resides in rural area (Table 1).

**Table 1:** Socio-demographic and economic characteristics of breastfeeding mothers in Goba woreda, Bale Zone, South East Ethiopia

Socio-demographic variables	Category	Number	Percent
**Age of mothers (years)**	15-19	43	7.1
	20-24	173	28.5
	25-29	219	36.0
	30+	173	28.5
**Religion**	Muslim	422	69.4
	Christian	186	30.6
**Ethnicity**	Oromo	542	89.1
	Amhara	60	9.9
	Other*	6	1.0
**Educational status of mothers**	Illiterate	139	22.9
	Able to read/Write	98	16.1
	Primary(1-8)	275	45.2
	Secondary and above(9+)	96	15.8
**Marital status**	Married	579	95.7
	Never married	10	1.7
	Other^†^	11	1.8
**Occupation of mothers**	House wife	498	81.9
	Farmer	42	6.9
	Business Woman**	25	4.1
	Student	15	2.5
	Employed	13	2.1
	Other^♠^	15	2.5
**Place of residence**	Urban	120	19.7
	Rural	488	80.3
**Sex of infant**	Male	318	52.3
	Female	290	47.7
**Age of infants**	< 6 months	283	46.9
	> 6 months	321	53.1

*Tigre, Gurage ^†^Widowed, divorced, separated ^♠ ^Daily laborer, house servants **A woman who is involved in some form of merchant or trade

Ninety nine percent of mothers had breastfeed at least once in their life time among which, 210(35.0%) of them squeezed out and discarded the colostrums and 104(17.2%) of mothers gave pre-lacteal food to their infants. In support of the idea of discarding colostrums, a 26 yrs old mother said *''... here in our communities, it is believed that colostrums(silga in Afan Oromo language) is not considered as a white milk, this might make the infant ill; therefore I discarded it out."*On the same issue a health extension worker from Aloshe kebele stated "...*mothers refused to give the first breast milk which they throw away because it may cause abdominal cramp*". With regard to the reason for pre lacteal feeding *a *29 years old rural mother of said *"...my infant's stomach cannot digest my breast milk; therefore I gave 'lega kibe'-meaning fresh butter to soften his stomach for the first two to three days."*

**Figure 1:**
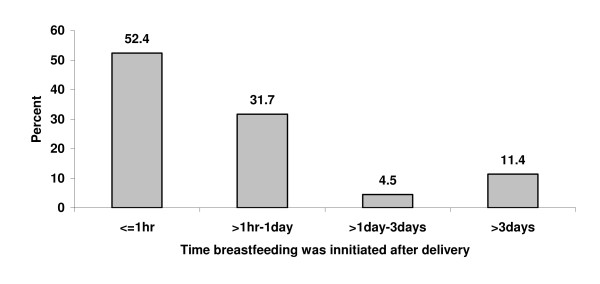
**Distribution of mothers by the time at which they initiated breastfeeding in Goba Woreda, South East Ethiopia**.

Among mothers who had ever breastfed, more than half 314(52.4%) of them initiated breastfeeding within one hour after delivery and 191(31.7%) initiated breastfeeding within the period of 1 hour to 1 day. Eleven percent of the mothers initiated breastfeeding after three days (Figure 1). Regarding timely initiation of breastfeeding, a mother with 20 days infant from Aloshe kebele made the following remark, *"...As usual most mothers initiate breastfeeding after one and half hour. I initiated breastfeeding after one hour because I had abdominal cramp." *Similarly, a health extension worker from the same kebele indicated;

"...Even if mothers initiated breastfeeding after one hour of delivery, most mothers who deliver in hospital do not breastfeed till they come to our health post which may take about three hours to walk".

### Factors associated with timely initiation of breast feeding

**Figure 2:**
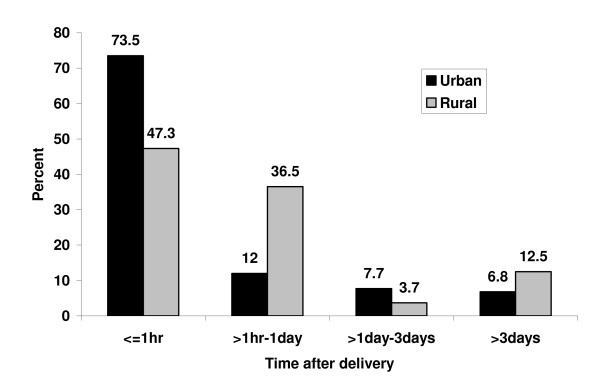
**Distribution of breastfeeding initiation by place of residence in Goba Woreda, South East Ethiopia**.

Timely initiation of breastfeeding was significantly associated with place of residence, institutional delivery, post natal advice on breast feeding and educational status. Urban mothers were more likely to initiate breastfeeding early as compared to their rural counterparts which was 73.5% and 47.3%, respectively (P < 0.001) (Table 2 and Figure 2). Among the obstetric and health service related factors, place of delivery and post natal information or advice on breastfeeding were significantly associated with timely initiation of breastfeeding (Table 3). Mothers who had formal education were 1.4 times as likely to initiate breastfeeding with in the first hour after delivery as compared to those mothers who had no formal education [OR:1.4 (95%C.I:1.03-2.03)]. Similarly, urban dwellers were 3 times more likely to practice timely initiation of breastfeeding when compared to their rural counterparts [OR: 3.1(95%C.I:1.98-4.84]).Mothers who delivered in health institutions were twice as likely initiate breastfeeding as compared to those delivered at their home[OR = 1.9(95%C.I:1.30-2.71)]).

**Table 2:** Breastfeeding initiation by socio-demographic characteristics of mothers in Goba Woreda, South East Ethiopia

Socio-demographic Variable		Time interval at which BF was initiated		
			
		≤ 1 hour N (%)	> 1 hour N (%)	P-value
**Age of mothers(year)**	15-19	23(53.5)	20(46.5)	0.356
	20-24	84(48.8)	88(51.2)	
	25-29	123(57.2)	92(42.8)	
	30-35	46(46.5)	53(53.5)	
	35+	38(54.3)	32(45.7)	
**Educational Status**	Illiterate	61(44.2)	77(55.8)	**0.025**
	Read/Write only	50(52.6)	45(47.4)	
	Primary(1-8)	148(54.6)	123(45.4)	
	Secondary and above (9+)	61(64.2)	34(35.8)	
**Marital status**	Married	300(52.6)	270(47.4)	0.730
	Never married	4(40.0)	6(60.0)	
	Other*	10(52.6)	9(47.4)	
**Occupation**	Employed	7(53.8)	6(46.2)	0.163
	Business Woman	16(66.7)	8(33.3)	
	Farmer	16(40.0)	24(60.0)	
	Student	10(66.7)	5(33.3)	
	House wife	260(52.8)	232(47.2)	
	Others**	5(33.3)	10(66.7)	
**Place of residence**	Urban	86(73.5)	31(26.5)	< 0.0001
	Rural	228(47.3)	254(52.7)	
**Sex of infant**	Male	164(51.9)	152(48.1)	0.851
	Female	150(53.0)	133(47.0)	

**Table 3:** Comparison of breastfeeding initiation by obstetric and health service related factors/practice in Goba Woreda, South East Ethiopia

Variable		Breastfeeding Initiation time		P-Value
		
	Category	≤ 1 hour N (%)	> 1 hour N (%)	
**Parity**	1	83(56.8)	63(43.2)	0.392
	2-4	152(50.3)	150(49.7)	
	5 and more	74(50.3)	73(49.7)	
**Birth interval(year)**	1 2-3	15(46.9) 139(53.5)	17(53.1) 121(46.5)	0.772
	4 and more	160(52.1)	147(47.9)	
**ANC visit**	Yes	270(53.1)	238(46.9)	0.218
	No	37(45.1)	45(54.9)	
**No of ANC visit**	1	11(44.0)	14(56.0)	0.574
	2-3	102(52.3)	93(47.7)	
	> = 4	157(54.5)	131(45.5)	
**Mother s counseled on BF at ANC**	Yes	134(56.8)	102(43.2)	0.150
	No	136(50.0)	136(50.0)	
**Place of delivery**	Home	202(48.1)	218(51.9)	**0.001**
	Health institution	106(63.9)	60(36.1)	
**Type of delivery**	Normal/Vaginal	298(53.5)	259(46.5)	0.099*
	C/S	6(33.3)	12(66.7)	
**Mothers counseled on BF at PNC**	Yes No	183(60.4) 131(44.3)	120(39.6) 165(55.7)	**< 0.0001**

ANC = Antenatal care, PNC = Post natal care, * Fisher exact test

Mothers who were counseled/advised on breastfeeding on postnatal were about 52% more likely to initiate breastfeeding within the first hour of delivery [OR: 0.52(95%C.I:0.38-0.72)] (Table 4). On multivariable regression analyses after adjusting for other variable in the model, place of residence and information/advice on breast feeding at post natal visit were isolated to be independent predictors of timely initiation of breastfeeding. Urban mothers were 4 times as likely to practice timely initiation of breastfeeding compared to their rural counterparts [OR: 4.1 [95%C.I: 2.31-7.30], P < 0.001. Similarly, mothers who got post natal education on breastfeeding were 2.7 times more likely to initiate breast feeding within one hour after delivery[OR: 2.7(95%CI:1.86-3.94)], P < 0.001 (Table 5).

**Table 4:** Bivariate and multivariable logistic regression showing factors associated with timely initiation of breastfeeding among mothers in Goba Woreda, South East Ethiopia

Variables	Timely initiation of breastfeeding			
	
	Yes	No	COR[95%C.I]	P-value
Attendance of formal education				
Yes	215(57.0)	162(43.0)	1.4[1.02-2.02]	0.038
No	98(48.0)	106(52.0)	1.0	
Residence				
Urban	86(73.5)	31(26.5)	3.1[1.98-4.84]	< 0.0001
Rural	228(47.3)	254(52.7)	1.0	
Place of delivery				
Home	202(48.1)	218(51.9)	1.0	
Health institution	106(63.9)	60(36.1)	1.9[1.30-2.71]	0.001
Post Natal Counseling				
Yes	183(60.2)	120(39.6)	0.52[0.38-0.72]	< 0.0001
No	131(44.3)	165(55.7)	1.0	

COR = Crude Odds Ratio; CI = Confidence Interval.

AOR = Adjusted Odds Ratio.

**Table 5:** Multivariable logistic regression showing predictors of timely initiation of breastfeeding among mothers in Goba Woreda, South East Ethiopia

Variable	Timely initiation of breastfeeding			
	
	Yes	No	AOR(95%C.I)	P value
Residence				
Urban	86(73.5)	31(26.5)	4.1[2.31-7.30]	< 0.0001
Rural	228(47.3)	254(52.7)	1.0	
Information/advise on BF at PNC				
Yes	183(60.2)	121(39.8)	2.7[1.86-3.94]	< 0.0001
No	132(43.4)	172(56.6)	1.0	

COR = Crude Odds Ratio; CI = Confidence Interval.

AOR = Adjusted Odds Ratio

## Discussion

This study attempted to determine the prevalence of timely initiation of breast feeding including factors associated with timely initiation of breastfeeding. Majority 602(98.7%) of mothers practiced ever breastfeeding. This figure is similar to the ever breastfeeding rate of the country (96%) and Oromia region (94.1%) and other regional states in Ethiopia ranging from 93% in Addis Ababa to 99% in Harari [8,17].

Although World Health Organization (WHO), global and national infant and young child feeding guidelines recommend that all newborns should start breastfeeding immediately (with in the first hour after delivery) and the feeding of colostrums be promoted, the current study showed that 35.0% of mothers squeezed and discarded the colostrums. This is due the cultural belief that colostrums can cause abdominal cramp in the child. The prevalence of pre-lacteal feeding in this study was 17.2% which is lower than the national pre-lacteal feeding reported by others (29%). The findings show that the reasons for giving pre-lacteal feeds are colostrums causes abdominal cramp, breast milk insufficiencies and to soften the stomach of the newborn. The prevalence of timely initiation of breastfeeding(52.4%) observed in the study area is better compared to the study report from Bangladesh where majority (88.8%) of the mothers initiated breastfeeding within three days after delivery and the prevalence of timely initiation of breastfeeding(41%) in Ghana [14].

The prevalence of timely initiation of breastfeeding in this study was relatively similar with studies done in Sudan (54.2%), Jordan (49.5%), Amhara region (60%) and Southern Nations Nationalities and Peoples (SNNP) region (50%). But, the figure we obtained is much lower than those observed in other studies from North Jordan (86.6%), Nepal (72.2%), Zambia; (70%), Bolivia (74%), Ethiopia (national) (69%), Oromia region (77%) [1,8-11,14,16-18].

The near average rate of timely initiation of breastfeeding in this study could possibly be attributed to the fact that in this study large proportion of respondents came from rural kebeles where timely initiation was less practiced (47.3% for rural and 73.5% for urban) and educational status is much lower in rural area as compared to urban. This can also be evidenced from the qualitative finding from Nurse, working at Goba hospital "...*as I told you ANC providers are very responsible for promoting breastfeeding. But I think health care professionals are not doing well especially in this regard".*

Significant proportion of mothers practiced pre lacteal feeding that is something other than breast milk (17.2%) which is lower than the Ethiopia Demographic Health Survey (EDHS) (29%). Binary logistic regression analysis showed no difference in the timing of initiation of breast feeding by gender of the infants. Other characteristics, such as attendance of formal education, being urban mothers, institutional delivery, and receiving post-natal advise/counseling on breastfeeding were associated factors of timely initiation of breastfeeding practices. In this study those mothers who gave birth in health institutions were better in practicing timely initiation of breastfeeding (48.1%Vs 63.9%). This is contrary to the result obtained from Ethiopian Demographic Health Survey which indicated that home delivery has direct relationship with timely initiation of breastfeeding [8]. However, in our analyses also, when the models is adjusted for other variables the effect of institutional delivery has disappeared which supports the findings of EDHS. The multivariable logistic regression analysis showed that, mothers from urban areas were 4 times more likely to initiate breastfeeding within one hour as compared to their rural counterparts. This result is consistent with the finding from Tanzania where timely initiation of breastfeeding was more common in urban areas (82%) than in the rural area (52%) [19]. But this finding was in contrast to a finding from Dominican Republic where initiation of breastfeeding was found to be slightly higher in rural areas than in urban areas where 95% and 92% respectively and the Ethiopian Demographic Health Survey finding where rural infants were more likely to breastfeed timely after birth than urban infants [8,20]. The difference in timely initiation of breastfeeding between urban and rural mothers or the difference within the same country might be explained by the fact that early initiation of breast feeding is more common among children whose mothers were assisted by trained traditional birth attendants and among children who are delivered at home. This was also indicated by the difference in the proportion of mothers who delivered at home and timely initiated BF in this study (48.1%) and in Ethiopian Demographic Health Survey (69.7%), [8]. In this study, counseling on breast feeding issues at post natal care period was an independent predictor of timely initiation of breastfeeding. Those mothers who got post-natal counseling on breastfeeding were 2.7 times more likely to initiate breastfeeding timely compared to mothers who did not receive counseling on breastfeeding after they gave birth. This might be related to the fact that mothers are exposed to breastfeeding information during their visit during the post natal period which is the most appropriate time for delivering key infant and young child feeding messages [1,15]

Ethiopia has developed National Infant and Young Child Feeding Guidelines with the objectives of achieving optimal breastfeeding. For the implementation, many health extension workers have been trained to provide community level breastfeeding promotion and in-service training has been given to promote breastfeeding at the health facility level. Despite all these interventions, the basic and easiest indicator of optimal breastfeeding (i.e. timely initiation of breastfeeding) remains at a near average. Furthermore, timely initiation is predicted by the nutrition behavior change communication delivered at during the post-natal period, implying the need for strengthening the delivery of key messages for optimal breast feeding through postnatal contacts both at the health institution and at health post levels.

The use of validated questionnaire and triangulating both quantitative and qualitative methods of data collection were the strengths of this study. However; a mother may have difficulty remembering when she initiated breastfeeding for her youngest infant; as a result, timely initiation of breastfeeding is subjected to potential recall bias. In addition, this study used a cross sectional study design; it is difficult to establish causal associations. Since the objective of this study is to assess the magnitude of the timely initiation and its determinants, we could not measure reason behind pre-lacteal feeding and colostrums removal quantitatively. Finally, this study is also limited to those missing data, thus interpretation of the finding shall take in to account the missing data.

## Conclusion

The study showed that the prevalence of timely initiation of breastfeeding was low. Pre lacteal feeding was common due to perceived traditional practices like, softening the stomach of the baby, breast milk insufficiencies, to avoid infant abdominal cramp. Maternal attendances of formal education, being urban resident and postnatal information/advice on breastfeeding were positively associated with timely initiation of breastfeeding. The factors associated with timely initiation should be taken into account while designing an intervention and, targeted, specific, and community oriented promotion of timely initiation of breastfeeding including women empowerment through education is recommended.

## Competing interests

The authors declare that they have no competing interests.

## Authors' contributions

TS conceived and designed the study, performed analysis and interpretation of data and drafted the manuscript. MG assisted with the design conception, analysis, and interpretation of data and the critical review of the manuscript. TB assisted the study design, data interpretation and critically reviewed the manuscript. All authors read and approved the final manuscript.

## Pre-publication history

The pre-publication history for this paper can be accessed here:

http://www.biomedcentral.com/1471-2458/11/217/prepub
